# Identifying clinically important COPD sub-types using data-driven approaches in primary care population based electronic health records

**DOI:** 10.1186/s12911-019-0805-0

**Published:** 2019-04-18

**Authors:** Maria Pikoula, Jennifer Kathleen Quint, Francis Nissen, Harry Hemingway, Liam Smeeth, Spiros Denaxas

**Affiliations:** 1 Institute of Health Informatics, University College London, 222 Euston Road, London, NW1 2DA UK; 2 Health Data Research UK London, University College London, 222 Euston Road, London, NW1 2DA UK; 3 Respiratory Epidemiology, Occupational Medicine and Public Health, National Heart and Lung Institute, Imperial College London, London, UK; 4 EHR Research Group, School of Hygiene and Tropical Medicine, London, UK

**Keywords:** COPD epidemiology, COPD exacerbations, Electronic health records, Cluster analysis

## Abstract

**Background:**

COPD is a highly heterogeneous disease composed of different phenotypes with different aetiological and prognostic profiles and current classification systems do not fully capture this heterogeneity. In this study we sought to discover, describe and validate COPD subtypes using cluster analysis on data derived from electronic health records.

**Methods:**

We applied two unsupervised learning algorithms (k-means and hierarchical clustering) in 30,961 current and former smokers diagnosed with COPD, using linked national structured electronic health records in England available through the CALIBER resource. We used 15 clinical features, including risk factors and comorbidities and performed dimensionality reduction using multiple correspondence analysis. We compared the association between cluster membership and COPD exacerbations and respiratory and cardiovascular death with 10,736 deaths recorded over 146,466 person-years of follow-up. We also implemented and tested a process to assign unseen patients into clusters using a decision tree classifier.

**Results:**

We identified and characterized five COPD patient clusters with distinct patient characteristics with respect to demographics, comorbidities, risk of death and exacerbations. The four subgroups were associated with 1) anxiety/depression; 2) severe airflow obstruction and frailty; 3) cardiovascular disease and diabetes and 4) obesity/atopy. A fifth cluster was associated with low prevalence of most comorbid conditions.

**Conclusions:**

COPD patients can be sub-classified into groups with differing risk factors, comorbidities, and prognosis, based on data included in their primary care records. The identified clusters confirm findings of previous clustering studies and draw attention to anxiety and depression as important drivers of the disease in young, female patients.

**Electronic supplementary material:**

The online version of this article (10.1186/s12911-019-0805-0) contains supplementary material, which is available to authorized users.

## Background

Chronic obstructive pulmonary disease (COPD) is responsible for considerable morbidity, mortality and health care expenditure worldwide. The estimated prevalence of COPD is about 1% in the general population and is increasing, projected by 2030 to be the third leading cause of death and the seventh leading cause of disability adjusted life years (DALYs) lost globally [1, 2]. Exacerbations of COPD are the second commonest cause of medical hospital admission in the UK with 8% of patients dying during an admission and 23% within a year of admission [3].

COPD is clinically heterogeneous [4]. Patients have different phenotypes with different aetiological and prognostic profiles and current disease classification systems do not fully capture this heterogeneity. Simple clinical measures such as the forced expiratory volume in one second (FEV_1_) and the number of acute exacerbations remain the best tools for disease staging as set out in the Global Initiative for Chronic Obstructive Lung Disease (GOLD) strategy [5]. However, it is increasingly recognised that treatment efficacy varies widely between individuals, and yet strategies are not informed by a detailed understanding of the underlying pathobiological mechanisms, therefore failing to alter the underlying causes of the disease but instead mostly address symptoms. There is an urgent need to identify, characterize and understand COPD subtypes in order to design, develop and evaluate more effective therapeutic strategies [6].

Previous COPD phenotyping studies have mostly focused on small, highly-selected populations with relatively short follow-up periods rather than population-based cohorts of COPD patients with extensive longitudinal information [7–12]. Typically, these studies underrepresent some demographic groups, such as female and GOLD 1 stage patients. The clinical markers included in these analyses exhibit great variation, and are often not collected in a primary care setting.

Using data from routinely collected electronic heath records (EHR) can potentially enable the identification of COPD subtypes that are representative of all COPD patients and provide higher-resolution longitudinal markers of comorbidity, disease severity and progression.

The objective of this study was to discover, describe and test the reproducibility of COPD subtypes by applying cluster analysis methods on large-scale EHR data. In order to assess the reproducibility of clusters, part of the data was set aside and only used to replicate the analysis findings.

## Methods

### Data sources

We selected anonymized patient EHR from the CALIBER resource described [13, 14] and validated [15] elsewhere. Briefly, the CALIBER resource is built on longitudinal structured records from three national sources for research: The Clinical Practice Research Datalink (CPRD), Hospital Episode Statistics (HES), and cause-specific mortality from the Office for National Statistics (ONS). CPRD provides anthropometric measurements, laboratory tests, clinical diagnoses, symptoms, prescriptions, and medical procedures, coded with the Read controlled clinical terminology. The primary care practices in CPRD and the subset of linked practices used in the present analysis are representative of the UK primary care setting [16] and have been validated for epidemiological research [17]. HES provides information about diagnoses (coded with the tenth revision of the International Classification of Diseases [ICD-10]) and medical procedures (coded with the 4th revision of the OPCS Classification of Interventions and Procedures) related to all elective and emergency hospital admissions across all National Health Service (NHS) hospitals in England. ONS provides a national mortality registry with physician-certified causes of death (coded using ICD-9 and ICD-10). All data sources were linked with a deterministic algorithm using patients’ NHS number (unique ten-digit identifier assigned at first interaction with the healthcare system), date of birth, sex and postcode which has been previously validated [17].

### Study population

**Fig. 1:**
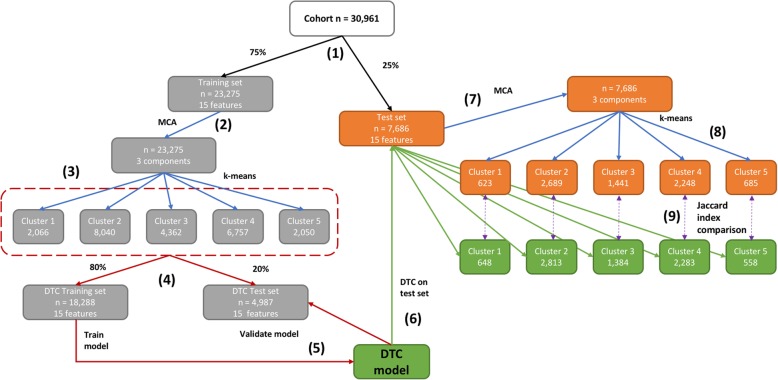
Main experiment steps (1) Split cohort into Training and Test sets; (2) Apply multiple correspondence analysis (MCA) to the Training set using all 15 potential cluster-generating features, results in 3 components; (3) Use 3 components derived in Step 2 from MCA analysis in k-means algorithm, results in k = 5 clusters; (4) Split Training set into a decision tree classifier (DTC) Training and DTC Test set to predict cluster labels obtained from k-means algorithm; (5) Train and validate DTC; (6) Apply DTC to Test set to predict cluster labels; (7) Apply MCA to Test set as in Step 2, results in 3 components; (8) Use 3 components derived in Step 7 from MCA analysis in k-means algorithm, results in k = 5 clusters; (9) Compare cluster assignments in Test set from Steps 6 and 8 by calculating the Jaccard Index (% of patients overlapping in the same cluster between the two solutions)

The study period was January 1st 1998 to January 3rd 2016, and individuals were eligible for inclusion if: a) they were (or turned) 35 years of age or older during the study period, b) they had been registered for at least one year in a primary care practice which met research data recording standards (known as Up To Standard and defined using CPRD algorithms examining patterns of data completeness and temporal gaps in recording) and c) had at least one diagnostic code for COPD. We did not impose an upper age limit. We used an open cohort design, so patients entered the study when they met the inclusion criteria. We set the index date for each participant to the date of the first COPD diagnostic code recorded in primary care while the participant was eligible. Patients were censored on the earliest date among the following: a) death from any cause (as defined in ONS or CPRD), b) leaving the primary care practice or c) the last practice data collection. In the analysis of all-cause mortality and cumulative hospitalisations, patient data derived from HES and ONS sources beyond the censoring date in the original analysis were used where available. Patients with missing baseline data were excluded from the analysis (Fig. 1).

### COPD definition

We have used validated algorithms and robust phenotyping approaches which have been evaluated and published previously. COPD diagnosis specifically was based on a validated algorithm (86.5% PPV) and used in over 50 publications [18] combined with either a current or former smoking status. The specific COPD definition READ codes are given in the Additional file 1 Given that the prevalence of COPD in never-smokers is less than 5 % in the UK, we did not include never-smokers, in order to minimise the chances of including patients who were misdiagnosed with COPD and to ensure alignment with UK clinical guidelines on the diagnosis of COPD by NICE [19]. Patients with a diagnosis of asthma were included, as it is possible to have both diseases and they co-occur in about 14% of patients with COPD [20].

### Cluster-generating features

The following baseline features used in generating the clusters were defined from the CPRD, recorded during primary care consultations: Body mass index (BMI), smoking status (current or ex), atopy, airflow obstruction as defined by Global Initiative for Chronic Obstructive Lung Disease (GOLD) stage [21]: 1 (FEV_1_% predicted > = 80%), 2 (50% < = FEV_1_% predicted < 80%), 3 (30% < = FEV_1_% predicted < 50%) and 4 (<= FEV_1_% predicted < 30%)– and eosinophil % of white blood cell counts, gastro-esophageal reflux disease (GERD), chronic rhinosinusitis (CRS), diabetes, anxiety, depression, ischemic heart disease (IHD), hypertension, heart failure. For sex, the value recoded in the patient demographic table by the general practitioner (GP) was used and for height we used the most recent value available.

We classified COPD therapy type with regards to different combinations of inhaled corticosteroids (ICS), Long Acting Muscarinic Antagonists (LAMA) and/or Long Acting Beta-2 Antagonists (LABA) as: a) no therapy (none of LAMA, LABA prescribed), b) mono-therapy (prescription of LABA or LAMA only), c) dual therapy (prescription of either LABA&LAMA or LABA&ICS or LAMA&ICS), and c) triple therapy: prescription of all LABA, LAMA and ICS.

Phenotyping algorithms for the covariates and comorbidities described here were defined using previously-published phenotyping algorithms from the CALIBER resource which have been used in over 60 publications using robust methodologies [15, 22–25].

### Supplementary variables

The following were supplementary variables, extracted from the EHR but not used in the generation of clusters: a) Age at index (diagnosis date) b) asthma diagnosis (stratified as pre−/post-COPD diagnosis or at any time), c) modified Medical Research Council (mMRC) dyspnoea scale, d) health utilisation quantified as the rate of consultations with clinical contact in the last year before index date and the last three years before the index date, e) deprivation. Socioeconomic deprivation, divided into quintiles, was measured using the Index of Multiple Deprivation (IMD), a neighbourhood deprivation score combining indices of unemployment, crime, income, education and other markers of social inequality [26].

### Clinically relevant events associated with cluster assignment

We used an a priori*-* specified set of clinically-relevant features to evaluate and interpret (but not to generate) clusters: a) rate of severe (resulting in a hospital admissions) or moderate (resulting in a primary care consultation) acute COPD exacerbation (AECOPD), b) respiratory and cardiovascular-related mortality and underlying cause of death. AECOPD recorded in primary and hospital care were identified using previously-validated phenotyping algorithms, using a combination of symptoms, antibiotics and oral corticosteroid prescriptions (excluding rescue packs) as well as codes for lower respiratory tract infections [3, 27]. All variables and outcomes are summarised in Additional file 1: Tables S1 and S2.

### Statistical methods

The main experiment has been outlined in Fig. 1. We randomly split the data into a training set (75%) and test set (25%). Statistical analyses described below were performed on the training data only unless otherwise specified. We performed multiple correspondence analysis (MCA [28]) using all covariates, transforming the three numerical covariates (BMI, FEV_1_% predicted and eosinophils) into categorical. This was deemed acceptable given that commonly used categories exist for BMI, COPD severity (indicated by the GOLD stage) and high eosinophil threshold. The MCA step resulted in a set of numerical components ranked by percentage of explained variance. The advantage of using the MCA components as input to the clustering algorithm instead of the original variables is that the components are continuous and orthogonal to each other as well as centred around zero with similar standard deviations. In particular, k-means requires continuous features on comparable scales so as to not be biased towards features with large value ranges. Using orthogonal (uncorrelated) features ensures that highly correlated variables do not dominate cluster assignments.

Clustering methods are a set of computational techniques that identify subsets in high-dimensional variable spaces by grouping according to their similarity. Such methods have been previously successfully applied to sub-phenotyping studies [12, 29]. We applied k-means and hierarchical clustering (HC) algorithms [30] on the numerical components resulting from the MCA step, using the distance between points in Euclidean space as the distance metric for both methods. Cluster-wise stability was assessed through resampling 30% of the training dataset 100 times and computing the Jaccard similarities [31] to the original clustering results. The Jaccard similarity or Jaccard index is a simple metric for the overlap of data-points between two clustering solutions. If a high proportion of patients are regularly clustered together the Jaccard similarity will be high.

We iteratively applied k-means clustering examining different values of k between 2 and seven and based the final choice of clusters on the average best silhouette coefficient [32]. Silhouette is a method of interpretation and validation of consistency within clusters. The technique provides a succinct graphical representation of how well each object lies within its cluster. The silhouette value is a measure of how similar an object is to its own cluster (cohesion) compared to other clusters (separation). A precise definition is given in the Additional file 1, section 3. The silhouette ranges from − 1 to 1, where a high value indicates that the object is well matched to its own cluster and poorly matched to neighbouring clusters. A negative value indicates that a data point would be more appropriately classified in its neighbouring cluster. The silhouette coefficient is calculated as the average silhouette of all the data in the dataset.

We used descriptive statistics to summarise and compare demographic characteristics, risk factors and clinical covariates within and between clusters and assigned cluster labels manually based on clinical input.

### Evaluation

Clusters were evaluated on the basis of results obtained from the training dataset. In order to test the reproducibility of the resulting five clusters on the test dataset, we trained a non-parametric decision tree classifier (DTC) [33], using the labels acquired from the clustering process, and validated the model on the remaining subset. More details on the DTC training process are included in the Additional file 1. In order to evaluate the robustness of our results, we repeated the experiment (MCA/k-means clustering) to the test set and compared the concordance of the acquired clusters with the cluster labels predicted by the DTC.

We obtained hazard ratios for the association between cluster label and time-to-CVD and respiratory-related mortality, adjusted for baseline age. We compared cumulative AECOPD between clusters.

Given the high levels of asthma misdiagnosis in this cohort, and in order to investigate the impact of removing all patients with potential asthma-COPD overlap, we performed a sensitivity analysis by repeating the analyses excluding all patients with diagnostic codes for asthma and compared our findings.

All analyses were performed using Python version 2.7 and relevant open-source libraries: scikit-learn, scipy, pandas and numpy.

## Results

### Cohort characteristics

**Fig. 2:**
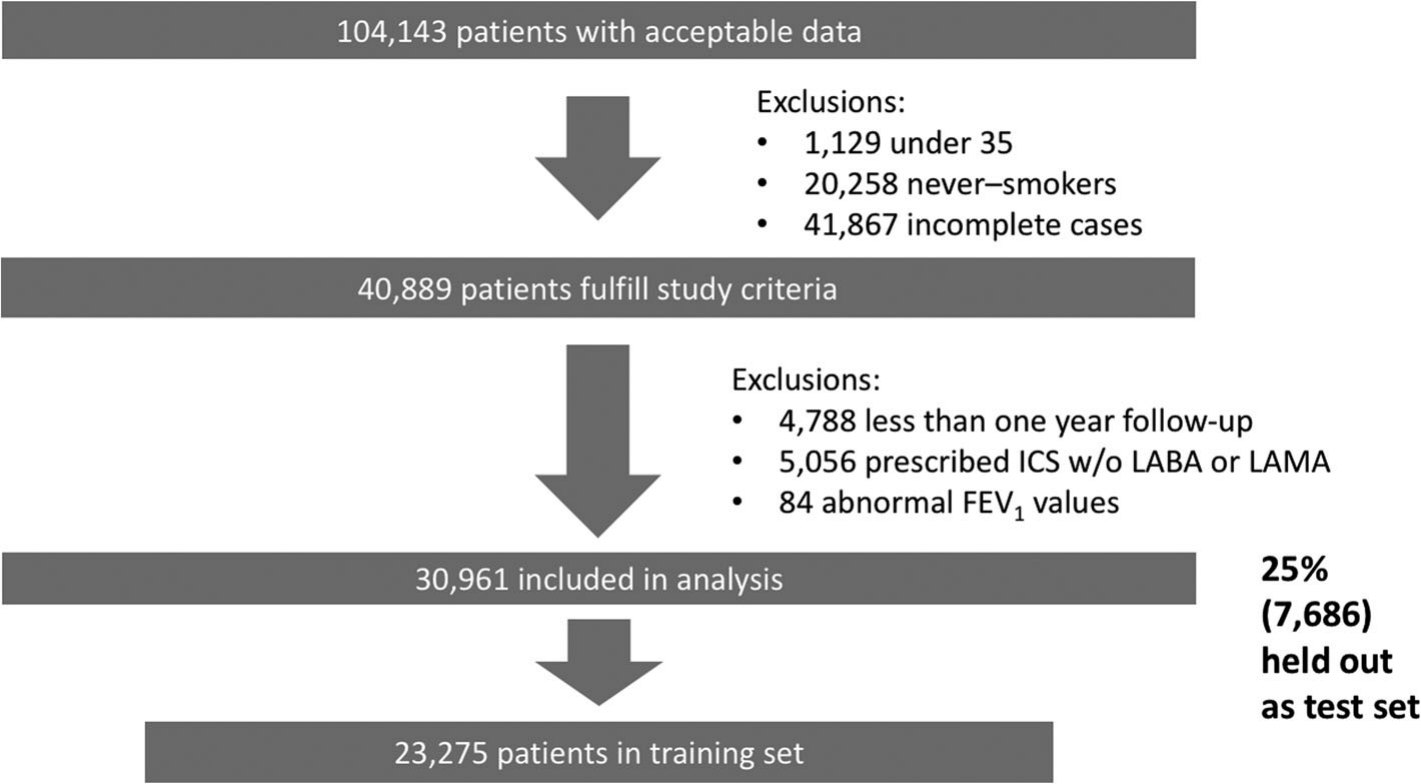
Patient flow diagram. Top level of excluded patient numbers not mutually exclusive. Second level of excluded numbers are given as applied sequentially

The study was comprised of 30,961 patients from 393 primary care practices contributing 146, 466 person-years of follow up. The inclusion and exclusion of patients in/from the study is described in Fig. 2.

The characteristics of the patients included overall as well as split by the training and testing datasets are shown in Table 1.

**Table 1:** Characteristics that were used the analysis: all patients (Entire cohort) and split by training and testing datasets^a^

Covariate	Level	Entire cohort	Training cohort	Test cohort
n		30,961	23,275	7686
Sex (male)	n (%)	16,885 (54.54)	12,723 (54.66)	4163 (54.15)
BMI	< 18.5	1305 (4.21)	978 (4.2)	327 (4.25)
	≥ 18.5, < 25	9926 (32.06)	7461 (32.06)	2465 (32.07)
	≥ 25, < 30	10,358 (33.45)	7758 (33.33)	2600 (33.83)
	≥ 30	9372 (30.27)	7078 (30.41)	2294 (29.85)
CRS	*n* (%)	590 (1.91)	445 (1.91)	145 (1.89)
Anxiety	*n* (%)	3123 (10.09)	2375 (10.2)	748 (9.73)
Atopy	*n* (%)	3809 (12.3)	2868 (12.32)	941 (12.24)
Depression	*n* (%)	3413 (11.02)	2605 (11.19)	808 (10.51)
Diabetes	*n* (%)	5001 (16.15)	3789 (16.28)	1212 (15.77)
Eosinophils > 2%	*n* (%)	20,363 (65.77)	15,299 (65.73)	5064 (65.89)
GERD		2759 (8.91)	2108 (9.06)	651 (8.47)
GOLD	1	8077 (26.09)	6017 (25.85)	2060 (26.8)
	2	15,536 (50.18)	11,749 (50.48)	3787 (49.27)
	3	6322 (20.42)	4730 (20.32)	1592 (20.71)
	4	1026 (3.31)	779 (3.35)	247 (3.21)
Heart failure	*n* (%)	4685 (15.13)	3579 (15.38)	1106 (14.39)
Hypertension	*n* (%)	10,515 (33.96)	7906 (33.97)	2609 (33.94)
IHD	*n* (%)	7134 (23.04)	5379 (23.11)	1755 (22.83)
Smoking	ex	14,447 (46.66)	10,920 (46.92)	3527 (45.89)
	current	16,514 (53.34)	12,355 (53.08)	4159 (54.11)
Therapy type	none	11,621 (37.53)	8775 (37.7)	2846 (37.03)
	mono	4071 (13.15)	3018 (12.97)	1053 (13.7)
	dual	10,261 (33.14)	7722 (33.18)	2539 (33.03)
	triple	5008 (16.18)	3760 (16.15)	1248 (16.24)

^a^*BMI* Body mass index, *CRS* Chronic rhinosinusitis, *GERD* Gastroesophageal reflux disease, *IHD* Ischaemic heart disease, *GOLD* Global initiative for chronic obstructive lung disease

#### Cluster analysis

MCA resulted in three numerical factors, the first two of which explained 89% of the variance. The factor loadings and contributions of each variable as well as a scatter diagram of the first two factors for each diagram are included in the Additional file 2 (variable loadings) and Additional file 1: Figure S1 respectively. We used the three first factors as input to the clustering algorithms.

**Fig. 3:**
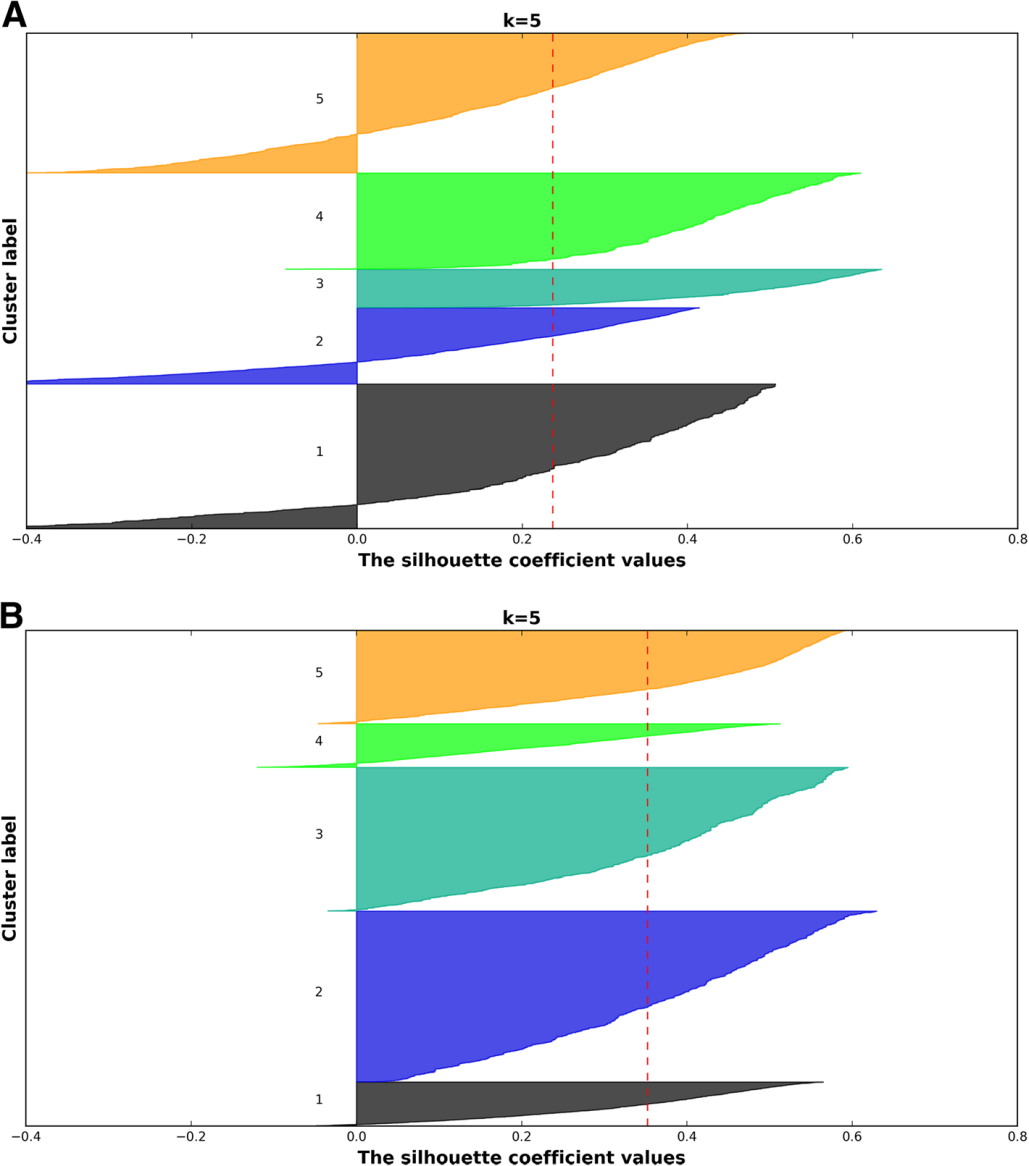
Silhouette plot of all samples resulting from the **a** HC 5 and **b** k-means cluster solutions. The dotted line represents the average silhouette score. Clusters are not annotated with specific labels at this stage

Both k-means and HC identified five clusters as the optimal number based on the silhouette criterion. The clusters obtained using the k-means algorithm had a higher average silhouette coefficient, with fewer negative samples, as shown in Fig. 3. Results obtained across both algorithms broadly displayed concordant patterns of clinical characteristics with regards to input and validation covariates. However, HC results tended to be unstable i.e. resampling the dataset produced significantly different results with regards to the optimal number of clusters and cluster membership. Conversely, in the case of k-means, after resampling 30% of the dataset 100 times and repeating both the MCA and k-means process, the Jaccard index, defined as the percentage of patients that are reassigned in the original clusters, was calculated at over 89%, showing adequate stability with regards to the original solution.

Table 2 contains the overall characteristics of patients belonging to the clusters in the training dataset. Subgroups were broadly labelled according to the dominant comorbidities of patients assigned to them as follows: Cluster 1 - Anxiety/Depression predominant, Cluster 2 - Non-comorbid predominant, Cluster 3 – Cardiovascular disease (CVD) / Diabetes predominant, Cluster 4 – Severe COPD/Frail predominant and Cluster 5 – Obesity/Atopy predominant.

**Table 2:**
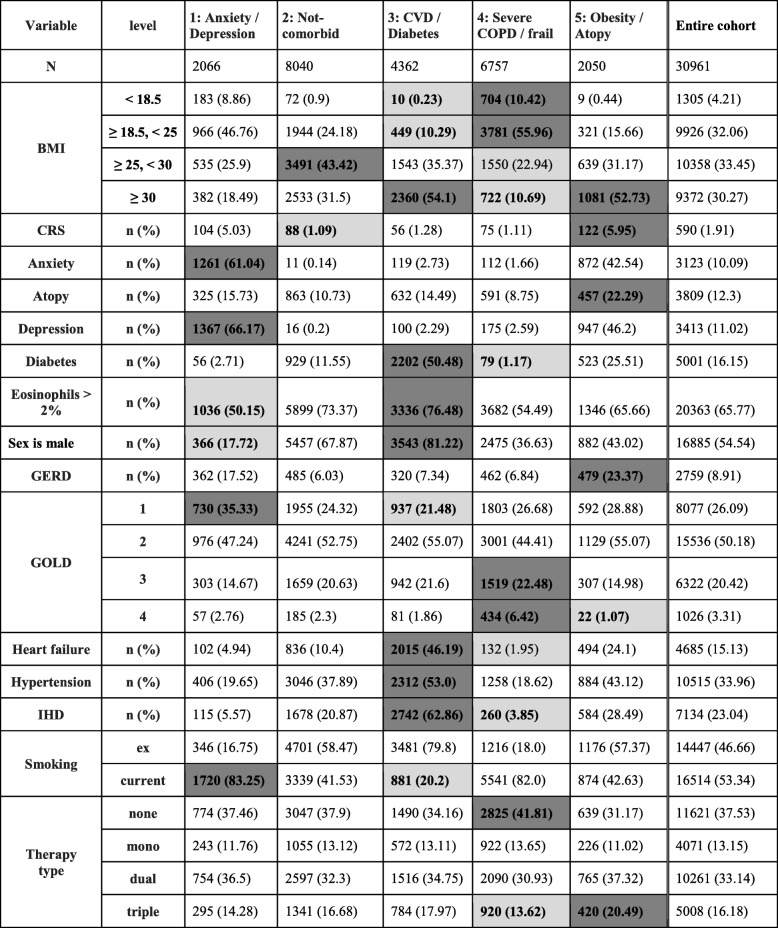
Characteristics of the 5 clusters identified by k-means clustering. Dark and light shading indicates higher and lower proportions respectively with regards to the entire cohort

The dark and light shading on the tables was done by clinical experts and it is intended to highlight clinically important information that clinicians would be immediately interested in. The shading also highlights the variables mostly drawn on by the clinicians when labelling the resulting clusters. A comparison of supplementary variables is shown in Table 3.

**Table 3:**
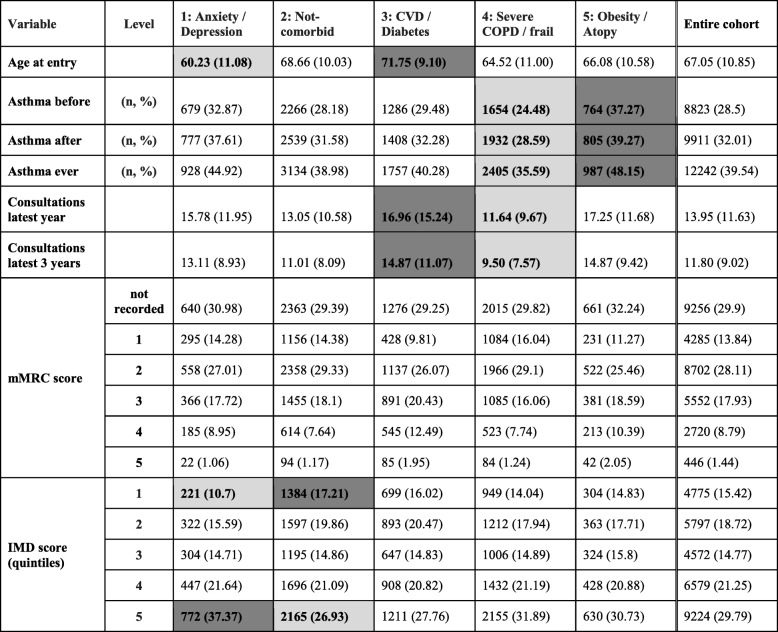
Variables not included as input in cluster analysis: Comparison between clusters. Higher IMD score values indicate more social deprivation (5th quintile is most deprived). Dark and light shading indicates higher and lower proportions respectively with regards to the entire cohort

#### Cluster 1 (anxiety/depression predominant)

Small cluster (9% of training set), composed predominantly of young, female smokers, with diagnoses for anxiety and depression, and an overall highest IMD score, with 37% of patients at the most deprived quintile.

#### Cluster 2 (non-comorbid predominant)

Largest cluster, predominantly male, with roughly equal numbers of current smokers and ex-smokers. Age at diagnosis slightly higher than the cohort average. The main feature of this cluster is low rates of most comorbidities. Most affluent (highest percentage in 1st IMD quintile).

#### Cluster 3 (CVD/ diabetes predominant)

Predominately male cluster of mostly ex-smokers with high incidence of IHD, heart failure, hypertension, high eosinophils, diabetes and highest BMI. Patients in this cluster are the oldest at the age of diagnosis compared to all other clusters.

#### Cluster 4 (severe COPD/frailty predominant)

Female, underweight/normal weight patients, mostly current smokers near the index date. Low prevalence of cardiovascular comorbidities and high eosinophil counts. The majority (56%) of all patients in the training set with severe disease as defined by GOLD stage IV airflow obstruction were assigned to this cluster.

#### Cluster 5 (obesity/atopy predominant)

Predominantly female cluster, small cluster, balanced between current smokers and ex-smokers. Highest asthma, CRS, GERD prevalence, second highest anxiety/depression and cardiovascular comorbidities, most atopic, mildest GOLD stages and overall high BMI, second only to cluster 3.

**Fig. 4:**
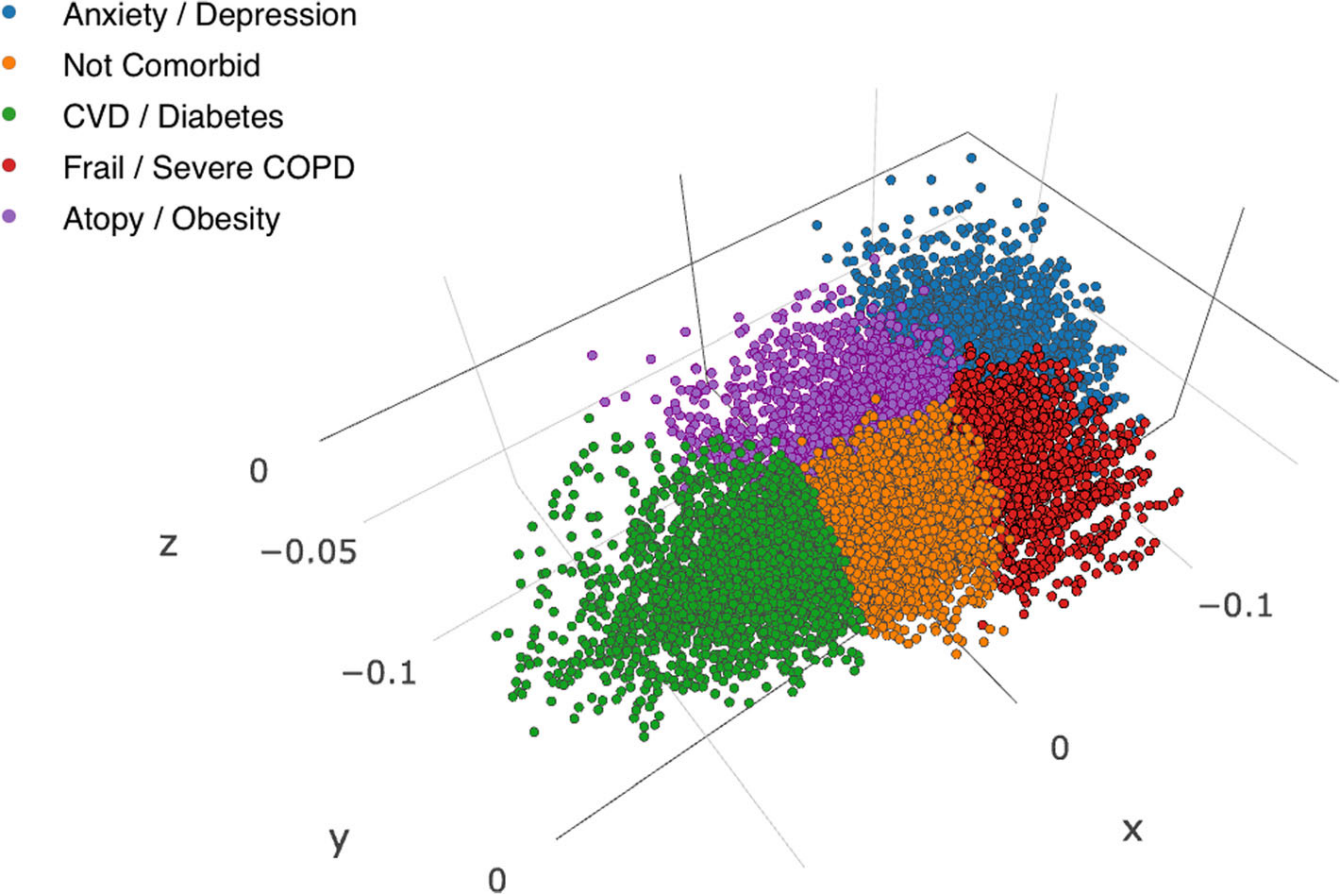
3D scatter plot of the three MCA Components colour-coded by cluster assignment

The final clustering solution can be graphically represented by plotting the three MCA components used in the analysis. A snapshot of a 3D representation is shown in Fig. 4. It is evident that the CVD / Diabetes shares no borders with the Anxiety / Depression or the Severe COPD clusters, whereas the Anxiety/Depression cluster shares no border with the Not-comorbid cluster. However, given this particular low-dimensional representation using the first three MCA components, it becomes clear from Fig. 3 that the clusters are not in any obvious way visually separable, and therefore k-means in combination with the Euclidean distance metric would do no more than segment the dataset attempting to minimise the distance from the assigned cluster centres.

### Prediction of cluster membership for unseen (test dataset) cases

**Fig. 5:**
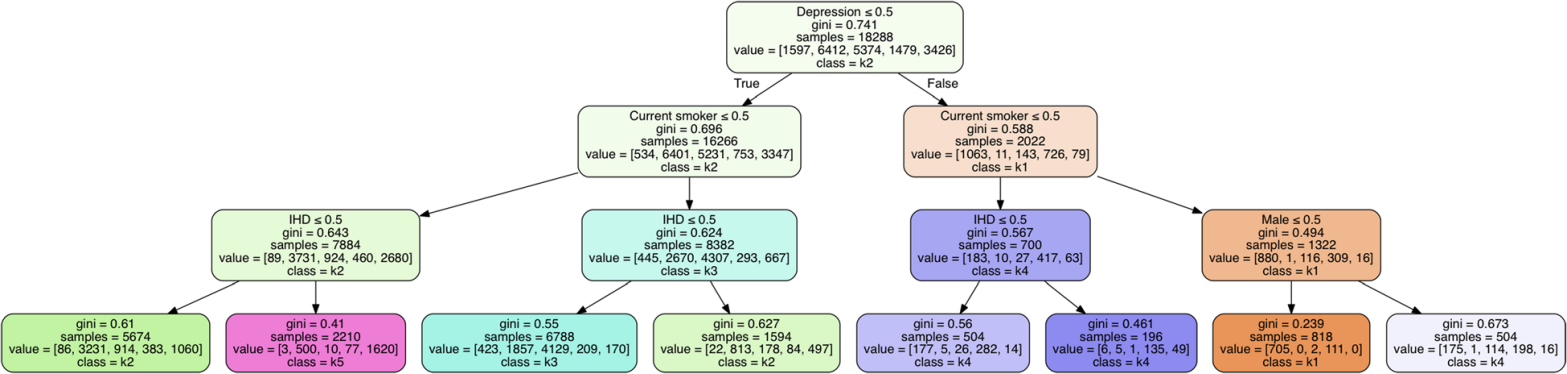
Simplified example output of decision tree classifier trained with a maximum depth of three

The DTC which was trained on 80% of the clustering training data with the cluster labels as the predicted classes and the same input of 15 variables as the input to the MCA. It was able to reach 96% accuracy in classifying unseen patients (the remaining 20%) to their respective cluster subgroup. An example graphical representation of the DTC output is shown in Fig. 5. We applied the DTC on the test set data and evaluated the performance of clustering on the clinical validation variables.

The characteristics of the five clusters derived from the test dataset were comparable to those of the training dataset with regards to variables included in the analysis as well as those that were not (Additional file 1: Tables S3-S5), indicating good reproducibility of the clusters in unseen data. We applied the full experiment separately on test data and found very good agreement between the two approaches with a 92% Jaccard index, meaning that the majority of patients are allocated to the same clusters in both the DTC and full MCA/k-means approaches.

### Outcomes

Mortality and AECOPD outcomes for all clusters are summarised in Table 4. Similarly, with previous studies, we observed in the overall cohort that the top three recorded underlying causes of mortality [34] (by ICD-10 chapters) were respiratory disease (30%) followed closely by cancer (29%) and circulatory disease (25%).

**Table 4:**
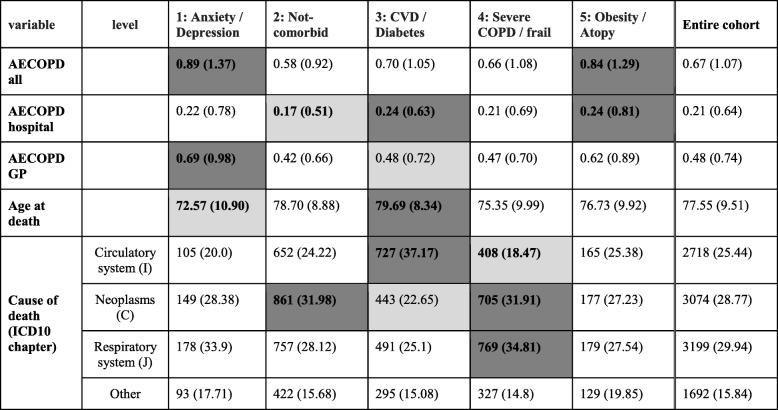
Mortality and AECOPD outcomes: Comparison between clusters. Dark and light shading indicates higher and lower proportions respectively with regards to the entire cohort

**Fig. 6:**
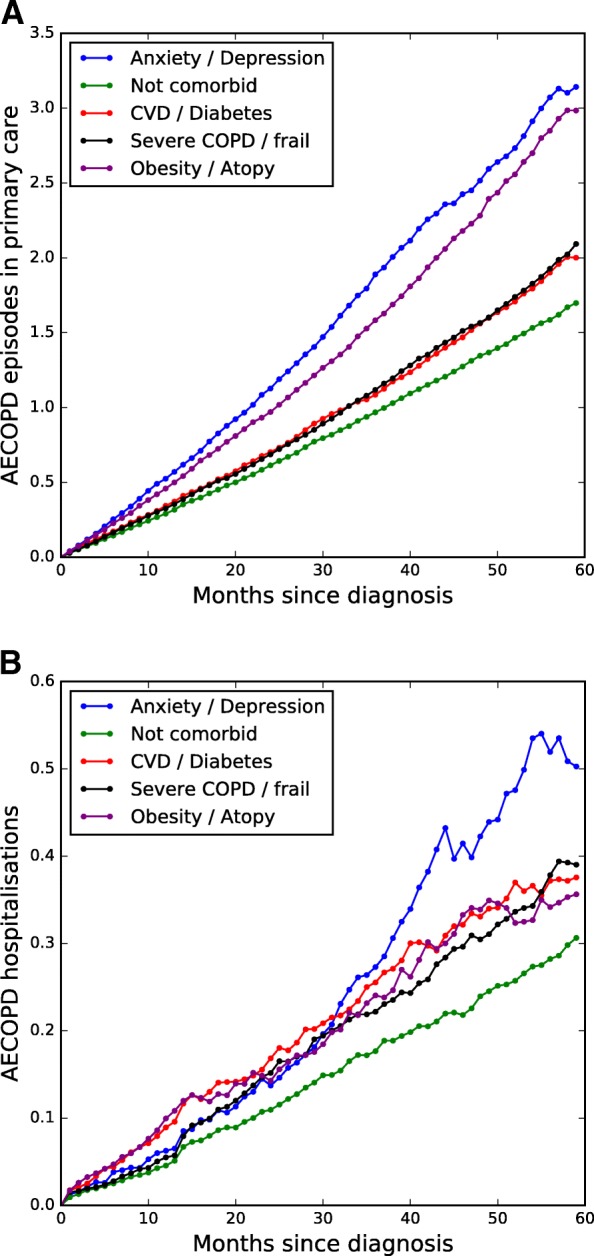
Cumulative AECOPD episodes by subgroup **a** in primary care and **b**) hospital admissions

Five-year cumulative number of AECOPD in primary care and AECOPD – related hospitalisations for all clusters were calculated and are shown in Fig. 6. The estimated relative hazard ratios for each subgroup resulting from the Cox proportional hazards regression on CVD and respiratory-related mortality are summarised in Table 5.

**Table 5:** Age-adjusted Cox regression with regards to CVD and respiratory related mortality

Characteristic	Hazard ratio
Age	1.08 [1.07–1.08]
Cluster	
Not comorbid	1
Anxiety / depression	1.28 [1.13–1.46]
CVD / diabetes	1.49 [1.38–1.60]
Severe COPD / frail	1.30 [1.20–1.40]
Atopy / obesity	1.15 [1.03–1.30]

#### Cluster 1 (anxiety/depression predominant)

Cluster 1 patients die younger on average and exhibit the highest rate of AECOPD among all other clusters, with 27% exacerbating at least once during the first year after diagnosis. Their hospitalisation rates are initially average but progress to an increasing rate (Fig. 6b). They are most likely to die from respiratory system diseases and are more at risk of death in comparison to all other clusters, with the exception of clusters 3 and 4.

#### Cluster 2 (non-comorbid predominant)

Age at death for cluster 2 is comparable to the cohort average. Their acute exacerbation rate is less than average, while 17% experience at least one exacerbation as recorded in primary care within the first year after diagnosis. Patients assigned to this cluster predominately die from cancer, whereas their CVD and respiratory mortality risk is the lowest out of all clusters.

#### Cluster 3 (CVD/ diabetes)

Patients in cluster 3 have the highest rates of AECOPD hospitalisation and are most likely to die from circulatory system diseases and least likely to die due to cancer or respiratory related causes. They have the highest average age at death.

#### Cluster 4 (severe COPD/frailty predominant)

Patients in cluster 4 show high rates of AECOPD hospitalisation and low rates of AECOPD in primary care, comparable to cluster 3. They are also most likely to die of cancer and respiratory related causes.

#### Cluster 5 (obesity/atopy predominant)

Patients in cluster 5 are second only to cluster 1 with regards to AECOPD exacerbations in primary care and 25% experience at least one AECOPD episode in the year after their diagnosis. They have on average comparable rates of AECOPD hospitalisations to cluster 3 and are most likely to die of respiratory causes.

### Sensitivity analyses

Patients excluded due to missing values had comparable disease severity and prevalence of comorbidities (where available), had similar rates of asthma diagnosis and higher rates of exacerbation in primary and secondary care. They were also more likely to die from respiratory related causes (Additional file 1: Tables S6-S8).

A high proportion of patients in the cohort have been diagnosed with asthma either prior or subsequent to their COPD diagnosis. The majority of these diagnoses are expected to be misclassifications, however it is not generally possible to know whether it was the COPD or asthma that was incorrectly diagnosed. We therefore performed the analysis excluding all patients with at least one asthma diagnostic code. The analysis yielded an optimal solution of k = 4 clusters, with the atopic cluster not providing a strong enough signal to form a separate cluster. The remaining clusters were similar to the original k5 solution, with over 96% patients being categorised to the corresponding clusters in both analysis (Anxiety/Depression, CVD, Not-comorbid, Severe COPD/frail). Patients belonging to the atopic cluster in the main analysis were categorised primarily as either Anxiety/Depression patients (33%) or Not-comorbid (53%). The characteristics of the k4 solution on non-asthmatic patients are shown in Additional file 1: Table S9.

## Discussion

This study identified five COPD clusters using EHR from primary care. These clusters can be easily identified from EHR data collected during routine clinical care that offer longitudinal high-resolution information across disease states [35, 36]. Individuals within these different clusters have differing outcomes, underlining the importance of phenotyping individuals with this heterogeneous disease more carefully.

The patients most at risk of AECOPD seen in primary care were female, current smokers and have anxiety and/or depression (cluster 1). Although the patients in this cluster initially presented with a lower hospitalisation rate for exacerbations, they did have a rapid increase in hospitalisations over time. This is in keeping with our current understanding of AECOPD; that moderate events progress to more severe events over time [37]. This cluster also had lowest average age at death, highlighting the importance of primary care exacerbation events on disease mortality, and were most likely to die of their COPD. This may be an important group in which to target prevention of exacerbation events early on in the course of disease.

Patients in the obesity/atopy predominant cluster (cluster 5), whilst having similar primary care exacerbation rates to cluster 1 and comparable hospitalisation rates to cluster 3, did not appear to have a similar increase in hospitalisations for exacerbation events over time or an increase in mortality or lower age of death compared to other clusters, suggesting that perhaps there is something protective about an asthma diagnosis in this population. This may be related to patient motivation around disease understanding, healthcare interaction or disease management and is a phenomenon that has been seen in other studies [34]. It is possible that some of these patients have been misclassified as having COPD and that is what is driving the trend seen here. The sensitivity analysis showed that this cluster is likely not distinctly present when all patients with asthma are excluded, further supporting the argument that misclassification of these patients has occurred.

Those patients with comorbid cardiovascular disease and diabetes unsurprisingly had some of the highest rates of hospital admission for AECOPD and were most likely to die of CVD. Interestingly they had the highest age at time of death and this may be a reflection of the disease-modifying treatments that are available for CVD that do not exist to the same extent for COPD. This cluster also highlights the important role co-morbidity plays in disease burden and progression. Given that these patients form a distinct cluster, this provides an important reason to investigate the presence of CVD among a COPD population.

The cluster with patients at the second greatest risk of death were more likely to be female current smokers. Patients in this cluster were also highly likely to be hospitalised with an AECOPD. They were most likely to die of cancer or their COPD. This may be an important subgroup in which to screen for lung cancer.

Although therapy type was a covariate included in the clustering analysis, it did not significantly vary within clusters and was not identified by the MCA as a variable that contributes to the overall cohort variance. This is an indication that, at least in the time-window immediately before and after diagnosis, therapy decisions are likely reactive with regards to patients’ early signs of frequent exacerbations.

Two of the identified subtypes correspond well with the two most reproducible subtypes identified in the clustering reproducibility analysis [38], which compared clustering results across cohorts: namely (1) a cluster with severe airflow limitation and low BMI, labelled as Severe COPD/frail in this study and (2) a cluster with high cardiovascular comorbidities and high BMI, labelled as CVD / Diabetes cluster in this study.

Previous research identifying clusters in COPD patients has mostly focused on smaller more specific or severe populations [7, 8]. However, a recent study by Burgel et al., using data from several thousand well-characterised COPD patients followed for three years, similarly reported clusters of patients with low comorbidities, with severe airflow limitation and nutritional depletion, cardiovascular comorbidities, or obesity [12]. In order to assess its performance on primary care EHR data, we implemented their proposed simple algorithm. In doing so we had to discard 30% of our dataset due to high rates of missingness for the mMRC score variable. Inconsistencies between the mMRC score and FEV_1_% predicted as recorded in primary care, led to the classification results being significantly different to the original paper, with less than 1% of patients being categorised as cluster 4 (not comorbid but severe disease), as opposed to 11% according to the Burgel model and dataset. This is likely a consequence of the subjective nature of mMRC score as recorded in the primary care setting. Furthermore, severity plays a significant role in the Burgel model with the result that certain clusters can only contain specific GOLD stage patients, therefore for some patients there is less opportunity for their increasing risk to be picked up while they are at an earlier disease stage.

### Limitations

Whilst this study has many strengths, including the large sample size and ability to determine clusters in a primary care setting where the majority of COPD is managed in the UK, there are some limitations. Whilst we used a validated set of Read terms to identify an asthma diagnosis, and this was seen in clusters with high prevalence of atopy, even clusters with the fewest asthma diagnoses were still close to or above 30%, signifying high rates of misdiagnosis. Equally the fact that the eosinophils are not highest in this group suggests some misdiagnosis and this is a well-known problem in primary care [20]. Secondly, the graphical representation of patients (Fig. 4) shows that although some clusters are clearly separable from each other, with no overlapping boundaries, for others those boundaries are not quite clear. This is one of the reasons why it should be possible for complex patients to belong to more than one cluster. This clustering approach would require a method that allows for multiple cluster membership in order to better deal with patients that display temporal variability with regards to disease severity and progression, such as for example multimorbid subgroups that fall between cluster margins. That is not something we explored in this analysis. In addition, the heterogeneity of patients still remains even within defined clusters, most importantly the non-comorbid and severe COPD clusters and further investigation of the patients belonging to those clusters is required.

## Conclusions

We applied cluster analysis to EHR data routinely generated in primary care, demonstrating that a diagnostic tool could potentially be developed to distinguish between COPD subtypes without requiring specialized testing. The phenotypic depth of the data allows for further analysis of significant proportions of the resulting subgroups, with regards to additional covariates and disease progression. Further investigation on the within subgroup variation, as well as the evolution of patient clusters through time will be the subject of future work.

## Additional file

Additional file 1: Supplementary material including feature definition and supplementary tables (DOCX 116 kb)

Additional file 2: Variable loadings. Multiple correspondence analysis loadings on all variables (PDF 41 kb)

## Abbreviations

AECOPD: Acute exacerbation of COPD
BMI: Body mass index
COPD: Chronic obstructive pulmonary disease
CPRD: Clinical Practice Research Datalink
CRS: Chronic rhinosinusitis
CVD: Cardiovascular disease
DTC: Decision tree classifier
EHR: Electronic heath records
FEV_1_: Forced expiratory volume in one second
GERD: Gastro-oesophageal reflux disease
GOLD: Global Initiative for Chronic Obstructive Lung Disease
GP: General practitioner / general practice
HC: Hierarchical clustering
HES: Hospital Episode Statistics
ICD-10: International classification of diseases (10th edition)
ICD-9: International classification of diseases (9th edition)
ICS: Inhaled corticosteroids
IHD: Ischemic heart disease
IMD: Index of multiple deprivation
LABA: Long acting beta-2 antagonists
LAMA: Long acting muscarinic antagonists
MCA: Multiple correspondence analysis
mMRC: modified Medical Research Council (dyspnoea scale)
NHS: National Health Service
ONS: Office for National Statistics
