# Supplementary material for: Identifying clinically important COPD sub-types using data-driven approaches in primary care population based electronic health records
Source: BMC Med Inform Decis Mak. 2019 Apr 18;19:86. doi: 10.1186/s12911-019-0805-0 (PMC6472089; doi:10.1186/s12911-019-0805-0)
Supplement: Supplementary file 1 — Supplementary material including feature definition and supplementary tables (DOCX 116 kb) [file 12911_2019_805_MOESM1_ESM.docx]

**Supplementary Material: Identifying clinically important COPD sub-types using data-driven approaches in primary care population based electronic health records**

**1. Covariate and Outcome definitions**

**Table S1: Definitions of covariates included as input in cluster analysis. Index date: Date of COPD diagnosis.**

| **Variable** | **Type** | **Baseline measurement** |
| --- | --- | --- |
| Sex | Binary: male / female | N/A |
| BMI | Categorical defined from continuous measurements:  Underweight (BMI < 18.5)  Normal (18.5 < BMI < 25)  Overweight (25 < BMI < 30)  Obese (BMI >= 30) | +/- two years from index date |
| Smoking status | Binary:  Current smoker / ex-smoker | +/- two years from index date  If current smoker code older than two years categorised as ex-smoker |
| Atopy | Binary, defined using minimum two codes out of following diagnoses: atopy, allergic rhinitis, eczema  Binary: Yes / no | Up to 10 years before index date and 2 years after index date. |
| Gastro-oesophageal Reflux Disease (GERD) | Binary, based on CRS codes or minimum of two acute rhinitis codes less than six months apart:  Binary: Yes / no | +/- two years from index date |
| Chronic rhinosinusitis (CRS) | Binary: Yes/no | Up to 10 years before index date and 2 years after index date. |
| Depression | Binary: Yes / no | +/- two years from index date |
| Anxiety | Binary: Yes / no | +/- two years from index date |
| Hypertension | Binary, based on hypertension codes: Yes / no | +/- two years from index date |
| Ischaemic heart disease (IHD) | Binary: Yes / no | +/- two years from index date |
| Heart failure | Binary: Yes / no | any time before or + two years from index date |
| Diabetes | Binary: Yes / no | any time before or + two years from index date |
| Eosinophils > 2% WBC | Binary: Yes / no | +/- two years from index date |
| GOLD grade | Categorical (1 – 4) based on FEV_1_ % predicted | +/- two years from index date |
| Therapy type | Categorical based on prescriptions for the following:  Mono – therapy: LABA or LAMA  Dual therapy: LABA & LAMA or LABA & ICS or LAMA & ICS  Triple therapy: LABA & LAMA & ICS | +/- six months from index date |

**Table S2: Definitions of supplementary variables and outcomes. (Not included as input in cluster analysis)**

| **Variable** | **Type** | **Baseline measurement** |
| --- | --- | --- |
| Age at entry | Numerical | N/A |
| Asthma diagnosis before index date | Binary based on code recorded: Yes / no | Up to 10 years before index date |
| Asthma diagnosis after index date | Binary based on code recorded: Yes / no | Any point after index date |
| Asthma diagnosis at any point | Binary based on code recorded: Yes / no | At any point |
| Index of multiple deprivation (IMD) | Ordinal, quintiles | As recorded by the ONS |
| Age at Death | Numerical | Derived from the date of death recorded by the ONS or CPRD |
| Cause of death | Categorical, based on ICD10 chapter | Recorded by the ONS death registry and using the underlying cause of death. |
| AECOPD hospitalisations | Rate: number of episodes / years in cohort | Measured from HES hospital admission from index date throughout patient’s follow up |
| AECOPD primary care | Rate: number of episodes / years in cohort | Measured from index date throughout patient’s follow up |
| AECOPD all events (hospitalisations and primary care) | Rate: number of episodes / years in cohort | Measured from index date throughout patient’s follow up |
| Consultations latest year | Rate: Number of clinical contact consultations over the last year | Up to a year before index date |
| Consultations latest 3 years | Rate: Number of clinical contact consultations over a maximum of three of years. | Up to three years before index date |
| mMRC dyspnoea scale | Categorical: 1-5 | +/- two years from index date |

**2. Multiple Correspondence Analysis Results**

**Figure S1: Scatter diagram of factor scores for factors 1 and 2 for all variables excluding hypertension and therapy (low loadings). For binary variables, only positive values are shown.**

**
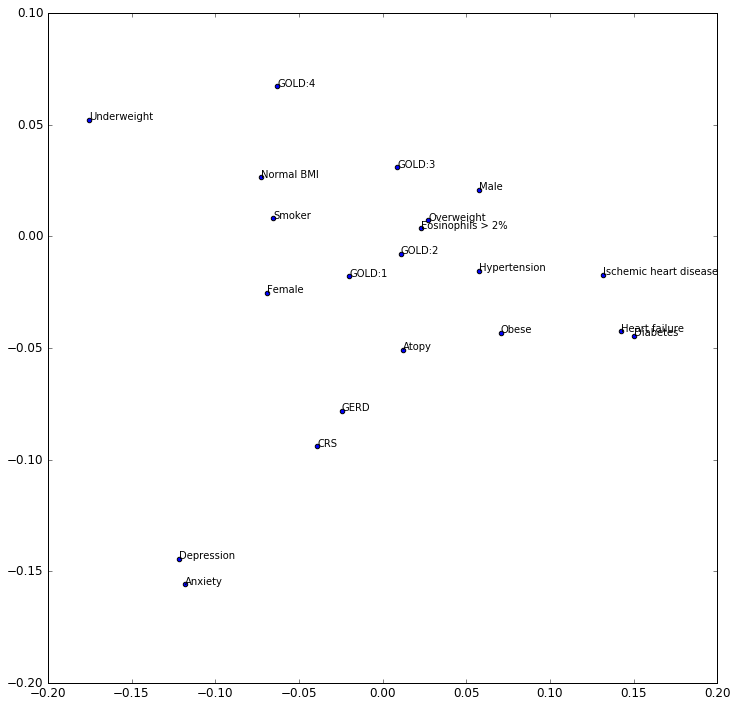
**

**3. Cluster analysis**

*Silhouette coefficient*

A silhouette for each data point is defined as:

*s ( i ) = b ( i ) – a ( i ) / max{a ( i ), b ( i )}*

Where for each data point *i*: *a ( i )* is the average distance of *i* with all other data within the same cluster and *b ( i )* is the lowest average distance of *i* to any other cluster, of which *i* is not a member.

The cluster with this lowest average dissimilarity is said to be the "neighbouring cluster" of *i* because it is the next best fit cluster for point *i*.

**4. Test set reproducibility of results**

**4.1 Training the DTC classifier**

In order to test the reproducibility of the resulting five clusters on the test dataset, we trained a non-parametric decision tree classifier (DTC)^30^ using the Gini impurity measure, using the labels acquired from the clustering process, and validated the model on the remaining subset. Data points with negative or zero silhouette values were not used in the training and assessing of the DTC. It is evident from Figure S3 that very few samples have negative or zero silhouette scores (1.7%), and these were the only ones excluding for the purpose of training the DTC classifier.

We then used the fitted model to obtain cluster labels for the test dataset and we also repeated the analysis pipeline on the test dataset and compared cluster assignment agreement between the fitted model and the clustering pipeline using the Jaccard index. There were no exclusions, on the basis of silhouette or otherwise, when reporting Jaccard index results for the testing set.

**Table S3: Characteristics of the five clusters identified on the test set using the decision tree classifier.**

| **variable** | **level** | **1: Anxiety / Depression** | **2: Not-comorbid** | **3: CVD / Diabetes** | **4: Severe COPD / frail** | **5: Obesity / Atopy** |
| --- | --- | --- | --- | --- | --- | --- |
| **n** |  | 648 | 2813 | 1384 | 2283 | 558 |
| **BMI** | **< 18.5** | 54 (8.33) | 19 (0.68) | 4 (0.29) | 245 (10.73) | 5 (0.9) |
|  | **≥ 18.5, < 25** | 308 (47.53) | 667 (23.71) | 139 (10.04) | 1265 (55.41) | 86 (15.41) |
|  | **≥ 25, < 30** | 159 (24.54) | 1244 (44.22) | 488 (35.26) | 523 (22.91) | 186 (33.33) |
|  | **≥ 30** | 127 (19.6) | 883 (31.39) | 753 (54.41) | 250 (10.95) | 281 (50.36) |
| **CRS** | **n (%)** | 33 (5.09) | 31 (1.1) | 27 (1.95) | 28 (1.23) | 26 (4.66) |
| **Anxiety** | **n (%)** | 399 (61.57) | 10 (0.36) | 39 (2.82) | 39 (1.71) | 261 (46.77) |
| **Atopy** | **n (%)** | 94 (14.51) | 317 (11.27) | 208 (15.03) | 216 (9.46) | 106 (19.0) |
| **Depression** | **n (%)** | 436 (67.28) | 10 (0.36) | 40 (2.89) | 70 (3.07) | 252 (45.16) |
| **Diabetes** | **n (%)** | 22 (3.4) | 332 (11.8) | 676 (48.84) | 31 (1.36) | 151 (27.06) |
| **Eosinophils > 2%** | **n (%)** | 351 (54.17) | 2070 (73.59) | 1031 (74.49) | 1232 (53.96) | 380 (68.1) |
| **Sex is male** | **n (%)** | 94 (14.51) | 1884 (66.97) | 1118 (80.78) | 825 (36.14) | 241 (43.19) |
| **GERD** | **n (%)** | 80 (12.35) | 186 (6.61) | 110 (7.95) | 146 (6.4) | 129 (23.12) |
| **GOLD** | **1** | 218 (33.64) | 692 (24.6) | 315 (22.76) | 628 (27.51) | 207 (37.1) |
|  | **2** | 307 (47.38) | 1442 (51.26) | 752 (54.34) | 1023 (44.81) | 263 (47.13) |
|  | **3** | 110 (16.98) | 600 (21.33) | 288 (20.81) | 514 (22.51) | 80 (14.34) |
|  | **4** | 13 (2.01) | 79 (2.81) | 29 (2.1) | 118 (5.17) | 8 (1.43) |
| **Heart failure** | **n (%)** | 28 (4.32) | 281 (9.99) | 615 (44.44) | 58 (2.54) | 124 (22.22) |
| **Hypertension** | **n (%)** | 143 (22.07) | 1049 (37.29) | 730 (52.75) | 428 (18.75) | 259 (46.42) |
| **IHD** | **n (%)** | 26 (4.01) | 598 (21.26) | 901 (65.1) | 80 (3.5) | 150 (26.88) |
| **Smoking** | **ex** | 97 (14.97) | 1604 (57.02) | 1124 (81.21) | 392 (17.17) | 310 (55.56) |
|  | **current** | 551 (85.03) | 1209 (42.98) | 260 (18.79) | 1891 (82.83) | 248 (44.44) |
| **Therapy**  **type** | **none** | 222 (34.26) | 1043 (37.08) | 478 (34.54) | 927 (40.6) | 176 (31.54) |
|  | **mono** | 85 (13.12) | 366 (13.01) | 204 (14.74) | 323 (14.15) | 75 (13.44) |
|  | **dual** | 233 (35.96) | 986 (35.05) | 462 (33.38) | 674 (29.52) | 184 (32.97) |
|  | **triple** | 108 (16.67) | 418 (14.86) | 240 (17.34) | 359 (15.72) | 123 (22.04) |

**Table S4: Variables not included as input in cluster analysis: Test set derived clusters.**

| **Variable** | **Level** | **1: Anxiety / Depression** | **2: Not-comorbid** | **3: CVD / Diabetes** | **4: Severe COPD / frail** | **5: Obesity / Atopy** |
| --- | --- | --- | --- | --- | --- | --- |
| **Age at entry** |  | 60.31 (11.42) | 68.65 (10.16) | 71.50 (9.26) | 64.35 (10.97) | 66.29 (10.75) |
| **Asthma before** | **(n, %)** | 213 (32.87) | 822 (29.22) | 386 (27.89) | 553 (24.22) | 200 (35.84) |
| **Asthma after** | **(n, %)** | 226 (34.88) | 906 (32.21) | 442 (31.94) | 672 (29.43) | 204 (36.56) |
| **Asthma ever** | **(n, %)** | 287 (44.29) | 1110 (39.46) | 543 (39.23) | 833 (36.49) | 258 (46.24) |
| **Consultations latest year** |  | 15.62 (11.23) | 12.84 (9.86) | 17.30 (13.34) | 11.57 (9.84) | 17.35 (12.99) |
| **Consultations latest 3 years** |  | 12.89 (8.56) | 10.81 (7.77) | 15.18 (10.97) | 9.51 (7.63) | 15.36 (10.59) |
| **mMRC score** | **not recorded** | 191 (29.48) | 846 (30.07) | 406 (29.34) | 678 (29.7) | 180 (32.26) |
|  | **1** | 93 (14.35) | 427 (15.18) | 147 (10.62) | 357 (15.64) | 67 (12.01) |
|  | **2** | 188 (29.01) | 821 (29.19) | 352 (25.43) | 646 (28.3) | 154 (27.6) |
|  | **3** | 121 (18.67) | 454 (16.14) | 294 (21.24) | 413 (18.09) | 92 (16.49) |
|  | **4** | 45 (6.94) | 224 (7.96) | 155 (11.2) | 166 (7.27) | 50 (8.96) |
|  | **5** | 10 (1.54) | 41 (1.46) | 30 (2.17) | 23 (1.01) | 15 (2.69) |
| **IMD score (quintiles)** | **1** | 79 (12.19) | 488 (17.35) | 232 (16.76) | 337 (14.76) | 82 (14.7) |
|  | **2** | 113 (17.44) | 545 (19.37) | 256 (18.5) | 398 (17.43) | 98 (17.56) |
|  | **3** | 82 (12.65) | 429 (15.25) | 208 (15.03) | 297 (13.01) | 80 (14.34) |
|  | **4** | 135 (20.83) | 573 (20.37) | 319 (23.05) | 520 (22.78) | 121 (21.68) |
|  | **5** | 239 (36.88) | 776 (27.59) | 369 (26.66) | 730 (31.98) | 177 (31.72) |

**Table S5: Mortality and AECOPD outcomes: Comparison between clusters in test set**

| **variable** | **level** | **1: Anxiety / Depression** | **2: Not-comorbid** | **3: CVD / Diabetes** | **4: Severe COPD / frail** | **5: Obesity / Atopy** |
| --- | --- | --- | --- | --- | --- | --- |
| **AECOPD all** |  | 0.75 (1.02) | 0.60 (1.04) | 0.71 (1.09) | 0.65 (1.02) | 0.81 (1.20) |
| **AECOPD hospital** |  | 0.19 (0.51) | 0.19 (0.60) | 0.24 (0.65) | 0.21 (0.63) | 0.23 (0.75) |
| **AECOPD GP** |  | 0.57 (0.78) | 0.42 (0.70) | 0.49 (0.77) | 0.46 (0.71) | 0.60 (0.83) |
| **Age at death** |  | 72.94 (11.47) | 79.23 (8.66) | 80.01 (8.01) | 75.50 (10.01) | 77.50 (9.10) |
| **Cause of death by ICD10 chapter** | Circulatory system (I) | 31 (17.42) | 244 (25.47) | 220 (36.42) | 123 (16.6) | 43 (25.29) |
|  | Neoplasms (C) | 49 (27.53) | 274 (28.6) | 127 (21.03) | 240 (32.39) | 49 (28.82) |
|  | Respiratory system (J) | 67 (37.64) | 288 (30.06) | 138 (22.85) | 288 (38.87) | 44 (25.88) |
|  | Other | 31 (17.42) | 152 (15.87) | 119 (19.7) | 90 (12.15) | 34 (20.0) |

**5. Sensitivity analyses**

**5.1 Excluded patients due to missing values**

Characteristics of all excluded patients due to missing values (In either BMI, eosinophil counts or FEV_1_ % predicted) are shown in Table S7. Variables not included in the analysis are presented in Table S8 and outcomes in Table S9.

**Table S6: Characteristics of excluded patients due to missing values and comparison with the analysis cohort.**

| **variable** | **Level** | **included** | **excluded** | **% missing values** |
| --- | --- | --- | --- | --- |
| **n** |  | 31902 | 34439 |  |
| **BMI** | **< 18.5** | 1355 (4.25) | 1236 (6.03) | 40.5 |
|  | **≥ 18.5, < 25** | 10256 (32.15) | 8162 (39.84) |  |
|  | **≥ 25, < 30** | 10643 (33.36) | 6402 (31.25) |  |
|  | **≥ 30, < 40** | 9648 (30.24) | 4685 (22.87) |  |
| **CRS** | **n (%)** | 602 (1.89) | 512 (1.49) |  |
| **Anxiety** | **n (%)** | 3250 (10.19) | 3087 (8.96) |  |
| **Atopy** | **n (%)** | 3888 (12.19) | 2817 (8.18) |  |
| **Depression** | **n (%)** | 3547 (11.12) | 3578 (10.39) |  |
| **Diabetes** | **n (%)** | 5113 (16.03) | 1896 (5.51) |  |
| **Eosinophils > 2%** | **n (%)** | 21062 (66.02) | 6757 (61.78) | 68.2 |
| **Sex is male** | **n (%)** | 17410 (54.57) | 18926 (54.96) |  |
| **GERD** | **n (%)** | 2843 (8.91) | 1977 (5.74) |  |
| **GOLD** | **1** | 8265 (25.91) | 2288 (20.65) | 67.8 |
|  | **2** | 16022 (50.22) | 5435 (49.06) |  |
|  | **3** | 6554 (20.54) | 2755 (24.87) |  |
|  | **4** | 1061 (3.33) | 601 (5.42) |  |
| **Heart failure** | **n (%)** | 4774 (14.96) | 4637 (13.46) |  |
| **Hypertension** | **n (%)** | 4657 (14.6) | 7725 (22.43) |  |
| **IHD** | **n (%)** | 6886 (21.58) | 5536 (16.07) |  |
| **Smoking** | **ex** | 15115 (47.38) | 17957 (52.14) |  |
|  | **current** | 16787 (52.62) | 16482 (47.86) |  |
| **Therapy**  **type** | **none** | 11989 (37.58) | 19801 (57.5) |  |
|  | **mono** | 4168 (13.07) | 2263 (6.57) |  |
|  | **dual** | 10591 (33.2) | 9875 (28.67) |  |
|  | **triple** | 5154 (16.16) | 2500 (7.26) |  |

**Table S7: Variables not included as input in cluster analysis: Comparison between full cohort and excluded patients**

| **Variable** | **Level** | **Included** | **Excluded** |
| --- | --- | --- | --- |
| **Age at entry** |  | 67.05 (10.85) | 66.30 (11.55) |
| **Asthma before** | **(n, %)** | 8823 (28.5) | 9075 (26.35) |
| **Asthma after** | **(n, %)** | 9911 (32.01) | 12727 (36.96) |
| **Asthma ever** | **(n, %)** | 12242 (39.54) | 15223 (44.2) |
| **Consultations latest year** |  | 13.95 (11.63) | 10.92 (10.30) |
| **Consultations latest 3 years** |  | 11.80 (9.02) | 9.25 (8.22) |
| **mMRC score** | **not recorded** | 9256 (29.9) | 25430 (73.84) |
|  | **1** | 4285 (13.84) | 2062 (5.99) |
|  | **2** | 8702 (28.11) | 3549 (10.31) |
|  | **3** | 5552 (17.93) | 1998 (5.8) |
|  | **4** | 2720 (8.79) | 1099 (3.19) |
|  | **5** | 446 (1.44) | 301 (0.87) |
| **IMD score (quintiles)** | **1** | 4775 (15.42) | 4630 (13.44) |
|  | **2** | 5797 (18.72) | 6185 (17.96) |
|  | **3** | 4572 (14.77) | 5251 (15.25) |
|  | **4** | 6579 (21.25) | 7328 (21.28) |
|  | **5** | 9224 (29.79) | 11015 (31.98) |

**Table S8: Mortality and AECOPD outcomes: Comparison between full cohort and training set**

| **variable** | **level** | **Included** | **Excluded** |
| --- | --- | --- | --- |
| **AECOPD all** |  | 0.67 (1.07) | 0.83 (1.27) |
| **AECOPD hospital** |  | 0.21 (0.64) | 0.28 (0.79) |
| **AECOPD GP** |  | 0.48 (0.74) | 0.57 (0.83) |
| **Age at death** |  | 77.51 (9.51) | 77.44 (9.76) |
| **Cause of death by ICD10 chapter** | Circulatory system (I) | 2718 (25.44) | 4373 (22.34) |
|  | Neoplasms (C) | 3074 (28.77) | 4765 (24.35) |
|  | Respiratory system (J) | 3199 (29.94) | 6704 (34.25) |
|  | Other | 1692 (15.84) | 3729 (19.05) |

**5.2 Analysis excluding all patients with asthma diagnosis**

The analysis pipeline was applied to a cohort free of asthma diagnoses (full cohort – no training/test split) which identified 4 clusters. The resulting cluster characteristics are summarised in Table S10.

**Table S9: Characteristics of the 4 clusters identified on the cohort free of asthma diagnoses**

| **variable** | **level** | **Not comorbid** | **Severe COPD / frail** | **Diabetes / CVD** | **Anxiety / depression** |
| --- | --- | --- | --- | --- | --- |
| **n** |  | 7229 | 5747 | 3605 | 2138 |
| **BMI** | **< 18.5** | 70 (0.97) | 670 (11.66) | 6 (0.17) | 151 (7.06) |
|  | **≥ 18.5, < 25** | 1711 (23.67) | 3316 (57.7) | 367 (10.18) | 942 (44.06) |
|  | **≥ 25, < 30** | 3031 (41.93) | 1292 (22.48) | 1354 (37.56) | 578 (27.03) |
|  | **≥ 30, < 40** | 2417 (33.43) | 469 (8.16) | 1878 (52.09) | 467 (21.84) |
| **CRS** | **n (%)** | 86 (1.19) | 50 (0.87) | 48 (1.33) | 134 (6.27) |
| **Anxiety** | **n (%)** | 251 (3.47) | 61 (1.06) | 173 (4.8) | 1269 (59.35) |
| **Atopy** | **n (%)** | 783 (10.83) | 429 (7.46) | 530 (14.7) | 291 (13.61) |
| **Depression** | **n (%)** | 286 (3.96) | 107 (1.86) | 172 (4.77) | 1325 (61.97) |
| **Diabetes** | **n (%)** | 960 (13.28) | 83 (1.44) | 1818 (50.43) | 127 (5.94) |
| **Eosinophils > 2%** | **n (%)** | 5087 (70.37) | 3078 (53.56) | 2727 (75.64) | 1154 (53.98) |
| **Sex is male** | **n (%)** | 4984 (68.94) | 2349 (40.87) | 3058 (84.83) | 611 (28.58) |
| **GERD** | **n (%)** | 584 (8.08) | 333 (5.79) | 300 (8.32) | 321 (15.01) |
| **GOLD** | **1** | 1853 (25.63) | 1450 (25.23) | 816 (22.64) | 731 (34.19) |
|  | **2** | 3880 (53.67) | 2504 (43.57) | 1985 (55.06) | 1092 (51.08) |
|  | **3** | 1351 (18.69) | 1452 (25.27) | 736 (20.42) | 278 (13.0) |
|  | **4** | 145 (2.01) | 341 (5.93) | 68 (1.89) | 37 (1.73) |
| **Heart failure** | **n (%)** | 881 (12.19) | 130 (2.26) | 1673 (46.41) | 159 (7.44) |
| **Hypertension** | **n (%)** | 2814 (38.93) | 1192 (20.74) | 1772 (49.15) | 607 (28.39) |
| **IHD** | **n (%)** | 1735 (24.0) | 235 (4.09) | 2415 (66.99) | 190 (8.89) |
| **Smoking** | **ex** | 3939 (54.49) | 926 (16.11) | 2783 (77.2) | 457 (21.38) |
|  | **current** | 3290 (45.51) | 4821 (83.89) | 822 (22.8) | 1681 (78.62) |
| **Therapy**  **type** | **none** | 3574 (49.44) | 2800 (48.72) | 1625 (45.08) | 1100 (51.45) |
|  | **mono** | 1235 (17.08) | 1004 (17.47) | 639 (17.73) | 390 (18.24) |
|  | **dual** | 1521 (21.04) | 1242 (21.61) | 800 (22.19) | 436 (20.39) |
|  | **triple** | 899 (12.44) | 701 (12.2) | 541 (15.01) | 212 (9.92) |

**Appendix**

**1. COPD Read terms used for case ascertainment in CPRD**

18476: COPD follow-up

45771: Chronic obstructive pulmonary disease does not disturb sleep

4084: Airways obstructn irreversible

794: Emphysema

998: Chronic obstructive airways disease

1001: Chronic obstructive pulmonary disease

5710: Chronic obstructive airways disease NOS

9520: Chronic obstructive pulmonary disease monitoring

9876: Severe chronic obstructive pulmonary disease

10802: Moderate chronic obstructive pulmonary disease

10863: Mild chronic obstructive pulmonary disease

10980: Centrilobular emphysema

11287: Chronic obstructive pulmonary disease annual review

14798: Emphysematous bronchitis

18621: Chronic obstructive pulmonary disease follow-up

18792: Chronic obstructive pulmonary disease monitoring admin

23492: Chronic bullous emphysema NOS

26018: Chronic obstructive pulmonary disease monitoring by nurse

26306: Chronic bullous emphysema

28755: Chronic obstructive pulmonary disease monitoring 1st letter

33450: Emphysema NOS

34202: Chronic obstructive pulmonary disease monitoring 2nd letter

34215: Chronic obstructive pulmonary disease monitoring 3rd letter

37247: Chronic obstructive pulmonary disease NOS

37371: Chronic obstructive pulmonary disease monitoring due

44525: Obstructive chronic bronchitis NOS

45998: Chronic obstructive pulmonary disease monitoring by doctor

93568: Very severe chronic obstructive pulmonary disease

12166: Other specified chronic obstructive airways disease

38074: Chronic obstructive pulmonary disease monitor phone invite

42258: Chronic obstructive pulmonary disease monitoring verb invite

42313: Health education - chronic obstructive pulmonary disease

45770: Chronic obstructive pulmonary disease disturbs sleep

45777: Chronic obstructive pulmonary disease clini management plan

**References**

1. Rousseeuw, P. J. Silhouettes: A graphical aid to the interpretation and validation of cluster analysis. *J. Comput. Appl. Math.* **20,** 53–65 (1987).
