# Supplementary material for: Identifying clinically important COPD sub-types using data-driven approaches in primary care population based electronic health records
Source: BMC Med Inform Decis Mak. 2019 Apr 18;19:86. doi: 10.1186/s12911-019-0805-0 (PMC6472089; doi:10.1186/s12911-019-0805-0)
Supplement: Supplementary file 2 — Variable loadings. Multiple correspondence analysis loadings on all variables (PDF 41 kb) [file 12911_2019_805_MOESM2_ESM.pdf]

Variable Loadings

variable	value	Factor score 1	Factor score 2	Factor score 3	Squared cosines 1	Squared cosines 2	Squared cosines 3	Contributions x 1000 1	Contributions x 1000 2	Contributions x 1000 3
BMI	1	-0.175169339	0.052189757	-0.097405377	0.71532046	0.063497264	0.221182277	38.57346838	11.94400182	162.3957424
BMI	2	-0.072360938	0.02660057	-0.007188246	0.873358667	0.118022864	0.008618469	50.21564786	23.67115195	6.747032915
BMI	3	0.027357872	0.007127	0.0227426	0.568529	0.038583495	0.392887505	7.463594887	1.766866811	70.22632226
BMI	4	0.070494236	-0.043062971	-0.003891372	0.726632205	0.271153616	0.00221418	45.21176121	58.85169703	1.875797331
GOLD stage	1	-0.020072677	-0.01784586	0.028158252	0.266076219	0.21031508	0.5236087	3.116192363	8.592022786	83.4951643
GOLD stage	2	0.010951669	-0.007812199	0.008972056	0.458715301	0.233415255	0.307869445	1.811319914	3.215051836	16.55216455
GOLD stage	3	0.008714714	0.031036324	-0.040645633	0.028219509	0.357918176	0.613862316	0.461743765	20.42876697	136.7599202
GOLD stage	4	-0.063048081	0.06721727	-0.106016751	0.201444592	0.228967379	0.569588029	3.980291264	15.78118682	153.2344326
Gender	0	-0.069095944	-0.025261026	0.003515877	0.880089735	0.117631554	0.00227871	64.75503465	30.19098122	2.282821498
Gender	1	0.057305698	0.02095059	-0.002915942	0.880089735	0.117631554	0.00227871	53.70550386	25.03931729	1.893290297
Smoking	0	0.074067525	-0.009149344	0.008618605	0.972007237	0.014831795	0.013160968	77.00376356	4.098670229	14.1960186
Smoking	1	-0.065464781	0.008086672	-0.007617578	0.972007237	0.014831795	0.013160968	68.05998365	3.622620712	12.54718925
Anxiety	0	0.013388502	0.017708898	0.002389835	0.35953342	0.629011162	0.011455417	4.815528356	29.38798693	2.089064578
Anxiety	1	-0.117818822	-0.155838306	-0.021030544	0.35953342	0.629011162	0.011455417	42.37664953	258.614285	18.38376829
Depression	0	0.015298124	0.018191609	0.002443369	0.409909467	0.579633954	0.010456579	6.217996359	30.67066135	2.159675084
Depression	1	-0.121386647	-0.1443457	-0.019387498	0.409909467	0.579633954	0.010456579	49.33818992	243.3637505	17.13646218
Eosinophils > 2%	0	-0.043527648	-0.007010372	-0.012815726	0.898774352	0.023313227	0.077912421	19.42447084	1.757550135	22.92669744
Eosinophils > 2%	1	0.022692759	0.003654796	0.006681367	0.898774352	0.023313227	0.077912421	10.12677818	0.916283409	11.95263342
Atopy	0	-0.001683287	0.007135593	-0.001903293	0.049386828	0.887472903	0.063140269	0.074323987	4.658861252	1.293780407
Atopy	1	0.011977277	-0.050772681	0.013542716	0.049386828	0.887472903	0.063140269	0.528845747	33.14971463	9.205779906
CRS	0	0.000763187	0.001833239	-0.000978298	0.118861404	0.685830304	0.195308292	0.017092359	0.344020754	0.382400206
CRS	1	-0.039154089	-0.094051351	0.050189982	0.118861404	0.685830304	0.195308292	0.876895656	17.64942428	19.61841957
Diabetes	0	-0.029238537	0.008651148	0.003365247	0.908435894	0.079529912	0.012034194	21.41251165	6.53898945	3.86212757
Diabetes	1	0.150367412	-0.044490966	-0.017306733	0.908435894	0.079529912	0.012034194	110.1198738	33.62859552	19.86207913
Hypertension	0	-0.029523044	0.007884106	0.001470626	0.931275061	0.066414152	0.002310787	17.21874659	4.283423333	0.581727788
Hypertension	1	0.057391812	-0.015326439	-0.002858847	0.931275061	0.066414152	0.002310787	33.47266839	8.326831925	1.130859395
Heart failure	0	-0.02591475	0.007705477	0.005601598	0.880953546	0.077885744	0.041160711	17.00222396	5.243454505	10.81612903
Heart failure	1	0.142614394	-0.04240488	-0.030826789	0.880953546	0.077885744	0.041160711	93.56686312	28.85584798	59.5234639
IHD	0	-0.039508696	0.005156171	0.005505663	0.964830528	0.016433129	0.018736344	35.90666131	2.133295668	9.493909798
IHD	1	0.131445925	-0.017154645	-0.018317408	0.964830528	0.016433129	0.018736344	119.4619094	7.09750126	31.58635615
GERD	0	0.002439475	0.007786075	-0.00266263	0.080786673	0.822970415	0.096242911	0.161914461	5.753563946	2.626339923
GERD	1	-0.024495429	-0.078182093	0.026736192	0.080786673	0.822970415	0.096242911	1.62582704	57.7730968	26.37179182
Therapy	0	-0.008189178	0.010493105	0.012602249	0.199600353	0.327709236	0.47269041	0.756417218	4.332071236	24.3900765
Therapy	1	0.003263532	0.011762451	-0.000477133	0.071368954	0.927105544	0.001525502	0.041316991	1.872213358	0.012024537
Therapy	2	0.000707873	-0.012036502	-0.001843225	0.003368049	0.973795738	0.022836214	0.004973642	5.016153466	0.459152028
Therapy	3	0.015038431	-0.009210161	-0.025242384	0.238519391	0.089464978	0.672015631	1.09301605	1.430087844	41.92938518
